# 

**Published:** 1860-07

**Authors:** 


### LIST OF COLLABORATORS.

ARMOR, S. G., M.D.,
Late Professor of Pathology and Clinical Medicine, Missouri Medical College.

ATKINSON, WM. B., M.D.,
Lecturer on Obstetrics, and Diseases of Women and Children, Philadelphia.

ATLEE, JOHN L., M.D.,
Late President Pennsylvania State Medical Society.

ATLEE, WALTER F., M.D.,
Philadelphia.

BARCLAY, ROBERT G., M.D.,
Jerusalem, Syria.

BELL, JOHN, M D.,
Philadelphia, formerly Professor of Medicine, Medical College of Ohio.

BEMISS, S. M., M.D.,
Professor of Clinical Medicine, University of Louisville.

BLACKBURN, L. P., M.D.,
Natchez, Miss.

BLACKIE, GEORGE C., M.D.,
Professor of Botany, University of Nashville.

BLACKMAN, G. C., M.D.,
Professor of Surgery, Medical College of Ohio.

BOBBS, J. L., M.D.,
Formerly Professor of Anatomy, Indianapolis.

BOWEN, W. S., M.D.,
New York.

BOZEMAN, N., M.D.,
Montgomery, Ala.

BRADFORD, J. T., M.D.,
Augusta, Ky.

BRINTON, JOHN H., M.D.,
Lecturer on Operative Surgery, Philadelphia.

BUMSTEAD, FREEMAN J., M.D.,
New York.

CARNOCHAN, J. M., M.D.,
Professor of Surgery in the New York Medical College.

CHIPLEY, W. S., M.D.,
Professor of Medicine, Transylvania University.

CLAPP, A., M.D.,
New Albany, Indiana.

CLIFFE, D. B., M.D.,
Franklin, Tenn.

DA COSTA, J., M.D.,
Lecturer on the Practice of Medicine at the Philadelphia Medical Association.

DICKSON, SAMUEL H., M.D.,
Prof. of Medicine, Jefferson Medical College.

DUNGLISON, RICHARD J., M.D.,
Physician to the Burd Orphan Asylum, and to the Albion Society, Philadelphia.

DUPIERRIS, M., M.D.,
Havana, Cuba.

EDGAR, W. F., M.D.,
United States Army.

FARRAR, S. C., M.D.,
Jackson, Miss.

FENNER, C. S., M.D.,
Memphis, Tenn.

FISCHER, EMIL, M.D.,
Philadelphia.

FLINT, AUSTIN, M.D.,
Professor of Clinical Medicine, Auscultation, and Percussion, New Orleans School of Medicine.

GADBURY, W. Y., M.D.,
Benton, Miss.

GIBBS, O. C., M.D.,

Chautauque County, New York.

HALL, J. P., M.D.,
Glasgow, Ky.

HAMMOND, WM. A., M D.,
United States Army.

HARDIN, JOHN, M.D.,
Professor of Obstetrics, Kentucky School of Medicine, Louisville.

HARTSHORNE, EDWARD, M.D.,
Surgeon to the Pennsylvania Hospital.

HASKINS, E. B., M.D.,
Professor of Medicine, Shelby Medical College, Nashville, Tenn.

HENTZ, C. A., M.D.,
Marianna, Florida.

HEWSON, ADDINELL, M.D.,
One of the Surgeons to Wills Hospital for Diseases of the Eye, Philadelphia.

HOLLINGSWORTH, SAMUEL L., M.D.,
Formerly Editor of the Medical Examiner, Philadelphia.

JEWELL, WILSON, M.D.,
Philadelphia, formerly President of the Quarantine and Sanitary Convention.

KEATING, WM. V., M.D.,
Lecturer on Obstetrics and the Diseases of Women, at the Phila. Medical Association.

KERR, JOHN G., M.D.,
Canton, China.

KUNKLER, G. A., M.D.,
Madison, Ia.

LOGAN, BEN., M.D.,
Shreveport, La.

LOPEZ, A., M.D.,
Mobile, Ala., formerly President of the Alabama State Medical Society.

MEIGS, J. AITKEN, M.D.,
Professor of the Institutes of Medicine in the Medical Department of Penna. College.

MITCHELL, S. WEIR, M.D.,
Lecturer on Physiology at the Philadelphia Medical Association.

MOREHOUSE, GEORGE R., M.D.,
Philadelphia.

PACKARD, JOHN H., M.D.,
Philadelphia.

RAPHAEL, B. I., M.D.,
New York.

REEVE, J. C., M.D.,
Dayton, Ohio,

REEVES, JAMES E., M.D.,
Fairmount, Virginia.

RIVES, L. C., M.D.,
Formerly Professor of Midwifery, Medical College of Ohio.

SMITH, J. LAWRENCE, M.D.,
Professor of Chemistry, University of Louisville.

SNEED, W. C., M.D.,
Frankfort, Ky., formerly President of Kentucky State Medical Society.

SPILMAN, C. H., M.D.,
Harrodsburg, Ky., formerly President of Kentucky State Medical Society.

STORER, HORATIO R., M.D.,
Formerly one of the Physicians to the Boston Lying-in Hospital, Boston, Mass.

SUTTON, GEO., M.D.,
Aurora, Ia.

SUTTON, W. L., M.D.,
Georgetown, Ky., formerly President Kentucky State Medical Society.

THOMPSON, S., M.D.,
Albion, Ill., formerly President of the Illinois State Medical Society.

TODD, L. BEECHER, M.D.,
Lexington, Kentucky.

WILLIAMS, E., M.D.,
Cincinnati, Ohio.

### Bi-Monthly Bibliographical Record.

AMERICAN.

Japanese Botany: being a fac-simile of a Japanese Book; with Introductory Notes and Translations. Philadelphia: J. B. Lippincott & Co., 1860. (From the publishers.)

Therapeutics and Materia Medica; a Systematic Treatise on the Action and Uses of Medicinal Agents; including their Description and History. By Alfred Stillé, M.D., etc. 8vo., pp. 813–975. Philadelphia: Blanchard & Lea, 1860. (From the publishers.)

Woman's Home Book of Health. By J. Stainback Wilson, M.D., etc. 12mo., pp. 364. Philadelphia: J. B. Lippincott & Co., 1860. (From the publishers.)

Quarterly Report of the Transactions of the College of Physicians of Philadelphia. 8vo. Vol. iii. No. 8. Philadelphia, 1860. (From the Secretary.)

Caffeine as an Antidote in the Poisonous Effects of Opium. By Henry Fraser Campbell, M.D., etc. Pamphlet, pp. 12. Augusta, 1860. (From the author.)

The Claims and Positions of Physiology. An Anniversary Oration, delivered before the South Carolina Medical Association. By J. Dickson Bruns, M.D, etc. Pamphlet, pp. 15. Charleston, 1860. (From the author.)

Dr. Wells, the Discoverer of Anæsthesia. Pamphlet, pp. 15. New York, 1860. (From Mrs. Wells.)

Record of Private Practice; Mortuary Record of Troy for the years 1858 and 1859. By T. C. Brinsmade, M.D. Pamphlet, pp. 43. Albany, 1860. (From the author.)

Forty-third Annual Report of the State of the Asylum for the Relief of Persons Deprived of their Reason. Pamphlet, pp. 31. Philadelphia, 1860. (From J. H. Worthington, M.D.)

The Institutes of Medicine. By Martyn Paine, M.D., etc. Fifth edition, 8vo., pp. 1109. New York: Harper & Brothers, 1859. (From the author.)

FOREIGN.

De la Galvanisation par Influence Appliquée au Traitement des Déviations de la Colonne Vertébrale, des Maladies de la Poitrine, des Abaissements de l'Utérus, etc. Par le Dr. J. Seiler. 8vo., pp. 157. Paris: J. B. Ballière et Fils, 1860. (From the publishers.)

On the Diseases, Injuries, and Malformations of the Rectum and Anus; with remarks on Habitual Constipation. By T. J. Ashton. 8vo., pp. 292. From the third and enlarged English edition. Philadelphia: Blanchard & Lea, 1860. (From the publishers.)

A Practical Treatise on the Diseases of the Lungs; including the Principles of Physical Diagnosis. By Walter Hayle Walshe, M.D., etc. 8vo., pp. 468. A new American from the third English edition. Philadelphia: Blanchard & Lea, 1860. (From the publishers.)

On Obscure Diseases of the Brain and Disorders of the Mind; their incipient Symptoms, Pathology, Diagnosis, Treatment, and Prophylaxis. By Forbes Winslow, M.D., etc., etc. 8vo., pp. 721. London: Churchill, 1860.

Foundation for a new Theory and Practice of Medicine. By T. Inman, M.R.C.P., etc. 8vo., pp. 374. London: Churchill, 1860.

On the Radical Cure of Variocele by Subcutaneous Incision. By Henry Lee. Pamphlet, pp. 24. London: Churchill, 1860.

Traité Pratique des Maladies de la Peau et de la Syphilis. Par C. M. Gibert. Third edition, 2 vols., 8vo. Paris: H. Plon, 1860.

The Pathology and Treatment of Venereal Diseases; comprising the most recent Doctrines on the Subject. By John Harrison, M.R.C.S. Demi 8vo. London: Simpkin, Marshall & Co., 1860.

D'une Nouvelle Espèce de Tumeurs Bénignes des os, ou Tumeurs à Myéloplaxes. Par E. Nélaton, M.D., etc. 8vo. Paris: Delahays, 1860.

On Rheumatism, Rheumatic Gout, and Sciatica; their Pathology, Symptoms, and Treatment. By H. W. Fuller, M.D., etc. Third edition, 8vo. London: Churchill, 1860.

A Manual of Human Microscopic Anatomy. By Albert Kolliker. 8vo. London: J. W. Parker & Son, 1860.

A Concise System of Operative Surgery. By C. F. Maudner, F.R.C.S., etc. 8vo., Part i. London: Churchill, 1860.

